# Intentional Carrier Doping to Realize *n*-Type Conduction in Zintl Phases Eu_5−*y*_La*_y_*In_2.2_Sb_6_

**DOI:** 10.3390/ma12020264

**Published:** 2019-01-15

**Authors:** Jianwei Lin, Wanyu Lv, Yayun Gu, Kai Guo, Xinxin Yang, Jingtai Zhao

**Affiliations:** 1 School of Materials Science and Engineering, Shanghai University, Shanghai 200444, China; Schrolock@shu.edu.cn (J.L.); lvwanyu@i.shu.edu.cn (W.L.); guyayun@i.shu.edu.cn (Y.G.); jtzhao@shu.edu.cn (J.Z.); 2 State Key Laboratory of Advanced Special Steel, Shanghai University, Shanghai 200444, China

**Keywords:** Eu_5_In_2_Sb_6_, Zintl phase, *n*-type, thermoelectric properties

## Abstract

Due to the tunable electrical transport properties and lower thermal conductivity, Zintl phase compounds have been considered as a promising candidate for thermoelectric applications. Most Sb-based Zintl compounds exhibit essentially *p*-type conduction as result of the cation vacancy. Herein, *n*-type Zintl phases Eu_5−*y*_La*_y_*In_2.2_Sb_6_ has been successfully synthesized via controlling the vacancy defect combined with intentional electron doping. Excess of In would occupy the vacancy while La doping enables the electron to be the major carrier at the measured temperate range, realizing the *n*-type conduction for Eu_5−*y*_La*_y_*In_2.2_Sb_6_ (*y* ≥ 0.04). Meanwhile, the thermal conductivity of Eu_5−*y*_La*_y_*In_2.2_Sb_6_ reduces from 0.90 W/mK to 0.72 W/mK at 583 K derived from the La doping-induced disorder. The maximum thermoelectric figure of merit *zT* = 0.13 was obtained. This work firstly realizes the *n*-type conduction in Eu_5_In_2_Sb_6_, which sheds light on the strategy to synthesize *n*-type Zintl thermoelectric materials and promotes the practical applications of Zintl thermoelectric devices.

## 1. Introduction

With the rapid developments of the global economy and industrial production, energy crises and environmental pollution have been critical issues in these years. Thus, exploring renewable and alternative energy resources is imminent in the near future. Thermoelectric material is a kind of functional material, which can realize the conversion of heat power and electrical energy. Therefore, it can be utilized for electricity generation, which would play an important role in energy recovery and reuse. The efficiency of a thermoelectric material is generally characterized by the thermoelectric figure of merit, *zT* = *α*^2^*T*/*ρκ*, where *α* is Seebeck coefficient (μV/K), *T* is the absolute temperature (K), *ρ* is the electrical resistivity (mΩ·cm), and *κ* is the thermal conductivity (W/mK) [1,2,3,4,5]. In principle, ideal thermoelectric material demands small electrical resistivity, large thermopower (absolute value of Seebeck coefficient), and low thermal conductivity even though the electrical and thermal transport properties are coupled strongly.

In 1995, Slack proposed the concept of phonon glass-electron crystal (PGEC), which implies high-performance thermoelectric materials contain electron-transport and phonon-scattering building blocks [6]. In this case, the electrical and thermal transport properties can be decoupled at a certain degree with independent functional blocks. Filled skutterudite is a typical example to demonstrate the term of PGEC since the CoSb_3_ framework is responsible for electron transport while rare-earth ions positioned in the cage scatter the phonons to reduce the thermal conductivity [7]. It is important to note that the Zintl compounds are another ideal system for designing high-efficiency thermoelectric materials [8]. Normally, they are valence-precise semiconductors composed of electropositive cations and electronegative anionic frameworks. Zintl phase compounds usually show tunable electrical transport properties and lower thermal conductivity, such as YbZn_2_Sb_2_ [9], Sr_3_GaSb_3_ [10], BaZn_2_Sb_2_ [11], Yb_14_MnSb_11_ [12,13], and Yb_9_Mn_4.2_Sb_9_ [14,15], and thus attracting much attention in the thermoelectric community in these years. However, Sb-based Zintl phase compounds normally exhibit *p*-type conduction, deriving from the instinct cationic vacancy [9]. It is well-know that a high-efficiency thermoelectric device requires *p*-legs and *n*-legs with a high figure of merit, *zT*, to realize energy conversion. Therefore, designing *n*-type Zintl phase compounds is fundamentally critical for matching *p*-type legs. Herein, we firstly present the results of the synthesis and characterization of *n*-type Eu_5−*y*_La*_y_*In_2.2_Sb_6_. By controlling the vacancy defect and introducing electron doping, the majority carrier is changed from a hole to electron and thus *n*-type conduction was realized. The maximum thermoelectric figure of merit, *zT* = 0.13, was obtained at 583 K. 

## 2. Experimental Section 

In this work, the polycrystalline samples, Eu_5_In_2+*x*_Sb_6_ (*x* = 0, 0.06, 0.1, 0.2) and Eu_5−*y*_La*_y_*In_2.2_Sb_6_ (*y* = 0.02, 0.04, 0.06, 0.08), were synthesized by element combination solid-state reaction. Starting materials, Eu (purity 99.99%), Sb (purity 99.999%), In (purity 99.99%), and La (purity 99.9%), were weighed according to the stoichiometric ratio in an argon-filled glove box (MBRAUN, Garching, Germany) (O_2_ < 0.3 ppm, H_2_O < 0.3 ppm), and then were transferred into a boron nitride crucible. These assembles were enclosed in evacuated fused quartz tubes. Subsequently, all the samples were heated to 1173 K for 24 h with a heating rate of 1 K/min in a muffle furnace, followed by an annealing process of 973 K for 72 h, and cooled to room temperature. The resulting products were ground into powder with a particle size of around 50 μm in an argon-filled glove box. The obtained powders were densified by a vacuum hot-press furnace at 873 K for 15 min under a pressure of 40 MPa for the thermoelectric properties’ characterization. 

The as-obtained samples were structurally characterized by powder X-ray diffraction (PXRD) on a diffractometer (18KW D/max2200V, Rigaku, Japan) with Cu Kα (*λ* = 1.54178 Å) radiation at room temperature. Seebeck coefficient and electrical conductivity were simultaneously measured by ZEM-3 (ULVAC, Chigasaki, Japan) on a rectangular-bars sample. The thermal conductivity of samples can be obtained from the relationship, *κ* = *dC*_p_*D*, where *d* is the experimental density, *C*_p_ is the heat capacity, and *D* is the experimental thermal diffusivity. The thermal diffusivity coefficients (*D*) were collected in a pellet-shaped sample with a diameter of Φ10 mm and a thickness of 1–2 mm by using the laser flash diffusivity (NETZSCH LFA457, Selb, Germany). The density (*d*) was achieved by the Archimedes’ method. *C*_p_ was estimated by the model of Dulong and Petit described as *C*_p_ = 3*NR*/*M*, where *N* is the number of atoms per formula unit, *R* is the universal gas constant, and *M* is the molar mass.

## 3. Results and Discussion 

**Figure 1:**
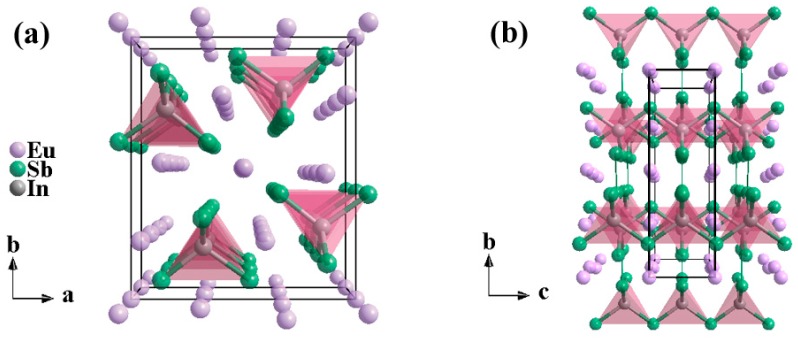
The crystal structure of Eu_5_In_2_Sb_6_ viewed along the *c* axis (**a**) and *a* axis (**b**).

The Zintl phase compound, Eu_5_In_2_Sb_6_, adopts the orthorhombic Ca_5_Ga_2_As_6_-type structure with the space group of *Pbam* (Figure 1) [16,17]. In this structure, every In atom is coordinated by four Sb atoms, forming a tetrahedron. These tetrahedra are connected by a sharing corner, thus producing one-dimension infinite (In_2_Sb_6_)^10−^ chains along the *c* axis. Furthermore, one-dimension ladder-like chains are bridged by Sb_2_ dumbbell groups. The rare earth element, Eu, between the chains provides electrons to the covalently-bonded anionic framework, leading to an overall charge balance described as [Eu^2+^]_5_[(4b)In^−^]_2_[(2b)Sb^−^]_4_[(1b)Sb^2−^]_2_ and satisfying the Zintl-Klemm concept. In fact, instinct cationic vacancy usually occurs in Sb-based Zintl compounds, such as EuZn_2_Sb_2_ [9]. For Eu_5_In_2_Sb_6_, it exhibits *p*-type conduction, which is probably derived from the cationic vacancy as well [18]. 

**Figure 2:**
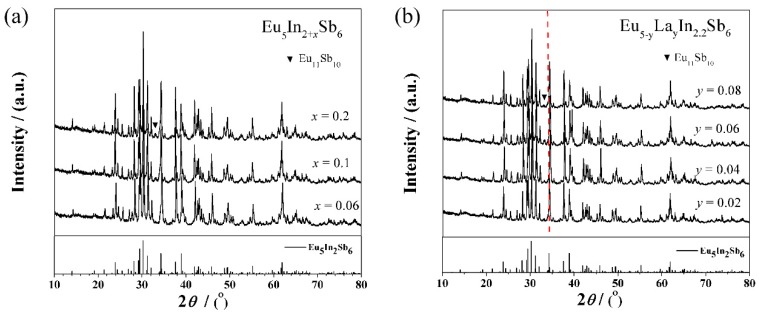
PXRD patterns for samples (**a**) Eu_5_In_2+*x*_Sb_6_ (*x* = 0, 0.06, 0.1, 0.2) and (**b**) Eu_5−*y*_La*_y_*In_2.2_Sb_6_ (*y* = 0.02, 0.04, 0.06, 0.08).

Figure 2 shows the PXRD patterns for the samples, Eu_5_In_2+*x*_Sb_6_ (*x* = 0.06, 0.1, 0.2) and Eu_5−*y*_La*_y_*In_2.2_Sb_6_ (*y* = 0.02, 0.04, 0.06, 0.08). The main reflections are consistent with the simulated pattern despite a trace of impurity of Eu_11_Sb_10_ in all samples. A similar phenomenon was also observed in our previous work [19]. Although the calculated lattice parameters do not follow the Regard’ law strictly, differences in electrical transport properties for the samples of Eu_5_In_2+*x*_Sb_6_ (*x* = 0.06, 0.1, 0.2) can be identified to demonstrate the occupation of In at lattice sites, which will be discussed below. For La-doped samples, the reflections’ peaks move in the higher-angle direction with increasing La content, indicating La^3+^ occupies Eu^2+^ successfully since Eu^2+^ (117 pm) shows a larger ionic radius compared with La^3+^ (103 pm) (Figure 2b) [19]. 

**Figure 3:**
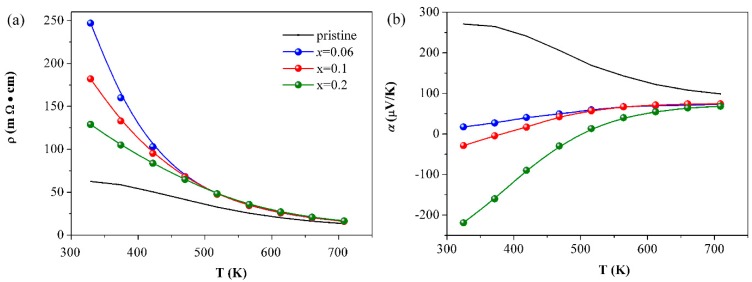
The resistivity (**a**) and Seebeck coefficients (**b**) as a function of the temperature for samples of Eu_5_In_2+*x*_Sb_6_ (*x* = 0, 0.06, 0.1, 0.2).

With the raise of the In content in Eu_5_In_2+*x*_Sb_6_ (*x* = 0, 0.06, 0.1, 0.2), the room-temperature resistivity increases and then decreases when *x* > 0.06, which is related to the varieties of the carrier type and concentration (Figure 3a). For the pristine Eu_5_In_2_Sb_6_ sample, it exhibits *p*-type conduction behavior due to the formation of the cation vacancy. The slight excess of In (*x* = 0.06) would trend to occupy the Eu vacancy, thus reducing the hole concentration. A greater In content would result in the major carrier changing from a hole to electron at room temperature, reflected by the sign of the Seebeck coefficients (Figure 3b). At the measured temperature range of 323–723 K, samples of Eu_5_In_2+*x*_Sb_6_ (*x* = 0, 0.06, 0.1, 0.2) display semiconductor behavior and the all resistivity approaches 12 mΩ·cm at 723 K, which indicates that a high temperature is favorable for the formation of the cation vacancy. In this case, *n*-type thermoelectrics are hard to achieve due to the electron-hole recombination. These results can be further understood from the temperature dependence of the Seebeck coefficients in Figure 3b. As mentioned above, nominal Eu_5_In_2_Sb_6_ is *p*-type semiconductor and the hole is dominant in the electrical transport properties. In the case of *x* = 0.06, the electron concentration is comparable to the hole concentration at room temperature. According to the formula, $\alpha =\frac{p\mu_{p}\alpha_{p-n\mu_{n}\alpha_{n}}}{p\mu_{p}+n\mu_{n}}$ (*n* and *p* are the electron and the hole concentrations, *μ_p_* and *μ_n_* are the electron and the hole mobility, and *α_p_* and *α_n_* are contributions of the electron and the hole to *α*), the contribution of the electrons and the holes to *α* compensated each other, resulting in the reduction of the Seebeck coefficients. A greater In content induces more electrons in the system and makes the electron become the major carrier. However, it is remarkable to note that the Seebeck coefficients of the samples of Eu_5_In_2+*x*_Sb_6_ (*x* = 0.1, 0.2) become positive since more cation vacancies form at higher temperatures. In this case, intentional electron doping is necessary to fabricate *n*-type Zintl phase thermoelectric materials.

**Figure 4:**
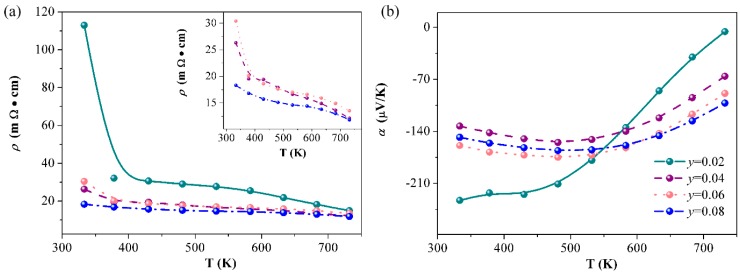
The resistivity (**a**) and Seebeck coefficients (**b**) as a function of the temperature for samples of Eu_5−*y*_La*_y_*In_2.2_Sb_6_ (*y* = 0.02, 0.04, 0.06, 0.08).

**Figure 5:**
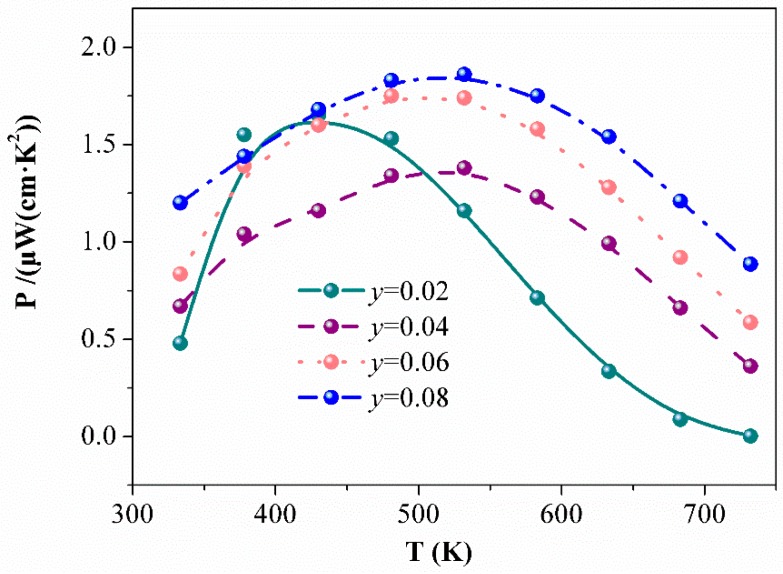
The power factor of Eu_5−*y*_La*_y_*In_2.2_Sb_6_ (*y* = 0.02, 0.04, 0.06, 0.08) depending on the temperature.

Samples of Eu_5−*y*_La*_y_*In_2.2_Sb_6_ (*y* = 0.02, 0.04, 0.06, 0.08) were synthesized with the aim to intentionally induce more electrons in the system. The resistivity and Seebeck coefficients depending on the temperature are shown in the Figure 4. As expected, La doping causes a decrease of resistivity for samples of Eu_5−*y*_La*_y_*In_2.2_Sb_6_ (*y* = 0.02, 0.04, 0.06, 0.08) (Figure 4a). When *y* = 0.02, the Seebeck coefficients approach zero at higher temperatures, indicating that the La content is insufficient (Figure 4b). A higher La content (*y* ≥ 0.04) leads to a negative and similar Seebeck coefficient at the measured temperature range. The power factors can be calculated from *PF*= *S*^2^/*ρ*, shown in Figure 5. The maximum value of 1.8 μW/(cm·K^2^) is obtained at 530 K when *y* = 0.08. 

**Figure 6:**
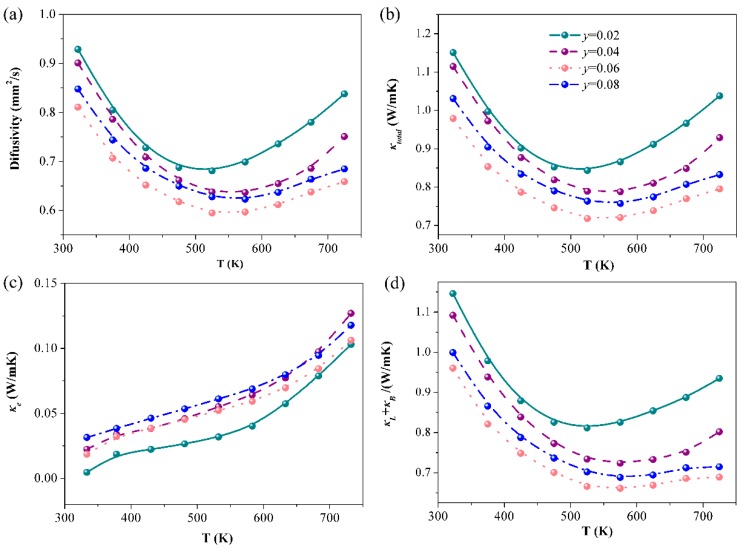
The thermal diffusion coefficient, *D* (**a**), the total thermal conductivity, *κ*_total_ (**b**), electronic thermal conductivity, *κ*_e_ (**c**), as well as bipolar and lattice thermal conductivity, *κ*_L_ + *κ*_B_ (**d**), as a function of the temperature for Eu_5−*y*_La*_y_*In_2.2_Sb_6_ (*y* = 0.02, 0.04, 0.06, 0.08).

The thermal diffusion coefficient (*D*), total thermal conductivity (*κ*_total_), electronic thermal conductivity (*κ*_e_), lattice, and bipolar thermal conductivity (*κ*_L_ + *κ*_B_) of La-doped samples are presented in Figure 6. All thermal diffusion coefficients are relatively low, ranging from 0.57–0.93 mm^2^/s, which is comparable to *p*-type Eu_5_In_2_Sb_6_ (Figure 6a) [18]. Therefore, the calculated total thermal conductivity is between 0.7 W/mK and 1.15 W/mK (Figure 6b). With increasing La contents, the total thermal conductivity decreases as result of the lattice disorder. 

**Figure 7:**
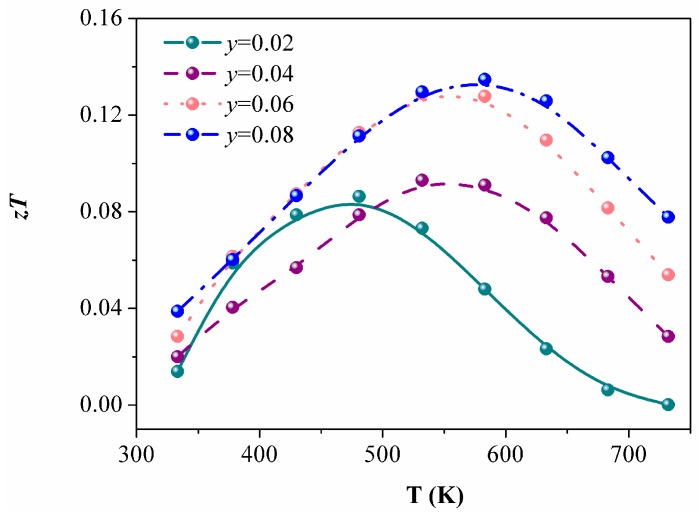
*zT* plots of Eu_5−*y*_La*_y_*In_2.2_Sb_6_ (*y* = 0.02, 0.04, 0.06, 0.08).

Figure 7 shows the figure of merit, *zT*, as a function of the temperature for La-doped samples. The peak, *zT*, for the Eu_4.92_La_0.08_In_2.2_Sb_6_ is 0.13 at 583 K. While the value is not as high as *p*-type Eu_5_In_2_Sb_6_, it is the first example to realize *n*-type conduction in the Zintl phase of Eu_5_In_2_Sb_6_, which provides an idea to obtain *n*-type thermoelectric materials by rational controlling of the defect and intentional electron doping.

## 4. Conclusions

By means of controlling the instinct defect and intentional electron doping, *n*-type Zintl phase compound, Eu_5−*y*_La*_y_*In_2.2_Sb_6_, was achieved. La doping can tune the carrier concentration and decease the thermal conductivity. The maximum thermoelectric figure of merit of 0.13 at 583 K was obtained, which was lower than the value in *p*-type Zn-doped Eu_5_In_2_Sb_6_. Even though the thermoelectric performance is not appealing, this is an example to demonstrate the strategy to synthesize *n*-type Zintl phase thermoelectric materials.

## References

[1] DiSalvo F.J. (1999). Thermoelectric cooling and power generation. Science.

[2] Sales B.C. (2002). Smaller is cooler. Science.

[3] Yang J., Caillat T. (2006). Thermoelectric materials for space and automotive power generation. MRS Bull..

[4] Chen G., Dresselhaus M.S., Dresselhaus G., Fleurial J.P., Caillat T. (2003). Recent developments in thermoelectric materials. Int. Mater. Rev..

[5] Snyder G.J., Toberer E.S. (2008). Complex thermoelectric materials. Nat. Mater..

[6] Slack G.A., Rowe D.M. (1995). CRC Handbook of Thermoelectrics.

[7] Nolas G.S., Morelli D.T., Tritt T.M. (1999). Skutterudites: A phonon-glass-electron crystal approach to advanced thermoelectric energy conversion applications. Annu. Rev. Mater. Sci..

[8] Kauzlarich S.M., Brown S.R., Snyder G.J. (2007). Zintl phases for thermoelectric devices. Dalton Trans..

[9] Zevalkink A., Zeier W.G., Cheng E., Snyder J., Fleurial J.P., Bux S. (2014). Nonstoichiometry in the Zintl phase Yb_1−δ_Zn_2_Sb_2_ as a route to thermoelectric optimization. Chem. Mater..

[10] Zevalkink A., Zeier W.G., Pomrehn G., Schechtel E., Tremel W., Snyder G.J. (2012). Thermoelectric properties of Sr_3_GaSb_3_ a chain-forming Zintl compound. Energy Environ. Sci..

[11] Yan R., Lv W., Wang K., Guo K., Yang X., Luo J., Zhao J.T. (2016). Enhanced thermoelectric properties of BaZn_2_Sb_2_ via a synergistic optimization strategy using co-doped Na and Sr. J. Mater. Chem. A.

[12] Brown S.R., Kauzlarich S.M., Gascoin F., Snyder G.J. (2006). Yb_14_MnSb_11_: New high efficiency thermoelectric material for power generation. Chem. Mater..

[13] Cox C.A., Toberer E.S., Levchenko A.A., Brown S.R., Snyder G.J., Navrotsky A., Kauzlarich S.M. (2009). Structure, Heat Capacity, and High-Temperature Thermal Properties of Yb_14_Mn_1−*x*_Al*_x_*Sb_11_. Chem. Mater..

[14] Bux S.K., Zevalkink A., Janka O., Uhl D., Kauzlarich S., Snyder J.G., Fleurial J.-P. (2014). Glass-like lattice thermal conductivity and high thermoelectric efficiency in Yb_9_Mn_4.2_Sb_9_. J. Mater. Chem. A.

[15] Ohno S., Zevalkink A., Takagiwa Y., Bux S.K., Snyder G.J. (2014). Thermoelectric properties of the Yb_9_Mn_4.2−*x*_Zn*_x_*Sb_9_ solid solutions. J. Mater. Chem. A.

[16] Park S.M., Choi E.S., Kang W., Kim S.J. (2002). Eu_5_In_2_Sb_6_, Eu_5_In_2__−*x*_Zn*_x_*Sb_6_: Rare earth Zintl phases with narrow band gaps. J. Mater. Chem..

[17] Chanakian S., Aydemir U., Zevalkink A., Gibbs Z.M., Fleurial J.P., Bux S., Snyder G.J. (2015). High temperature thermoelectric properties of Zn-doped Eu_5_In_2_Sb_6_. J. Mater. Chem. C.

[18] Lv W.Y., Yang C.H., Lin J.W., Hu X.Y., Guo K., Yang X.X., Luo J., Zhao J.T. (2017). Cd substitution in Zintl phase Eu_5_In_2_Sb_6_ enhancing the thermoelectric performance. J. Alloys Compd..

[19] Shannon R.D. (1976). Revised effective ionic radii and systematic studies of interatomic distances in halides and chalcogenides. Acta Crystallogr. A.

